# An Electromagnetic Vibration Energy Harvester with a Tunable Mass Moment of Inertia

**DOI:** 10.3390/s21165611

**Published:** 2021-08-20

**Authors:** Peter Ibrahim, Mustafa Arafa, Yasser Anis

**Affiliations:** 1 Mechanical Design and Production Department, Faculty of Engineering, Cairo University, Giza 12613, Egypt; Pei012@student.bham.ac.uk (P.I.); yanis@eng.cu.edu.eg (Y.A.); 2 Mechanical Engineering Department, American University in Cairo, New Cairo 11835, Egypt

**Keywords:** variable inertia, energy harvesting, frequency tuning, self-tuning

## Abstract

This paper presents a vibration-based electromagnetic energy harvester whose resonance frequency can be adjusted to match that of the excitation. Frequency adjustment is attained by controlling a rotatable arm, with tuning masses, at the tip of a cantilever-type energy harvester, thereby changing the effective mass moment of inertia of the system. The rotatable arm is mounted on a servomotor that is autonomously controlled through a microcontroller and a photo sensor to keep the device at resonance for maximum power generation. A mathematical model is developed to predict the system response for different design parameters and to estimate the generated power. The system is investigated analytically by a distributed-parameter model to study the natural frequency variation and dynamic response. The analytical model is verified experimentally where the frequency is tuned from 8 to 10.25 Hz. A parametric study is performed to study the effect of each parameter on the system behavior.

## 1. Introduction

The development of vibration-based energy harvesters has been a subject of considerable research in the past decade. The ubiquity of mechanical vibration as a source of energy for low-power devices has attracted attention, especially with the deployment of wireless sensors and self-powered devices. Despite the availability of such an energy source, challenges still remain to develop efficient energy harvesters that are well-suited to the nature of the available vibration environment.

One of the challenges is encountered in inertial devices, which are typically mounted on the surfaces of vibrating machines, structures, etc. for energy harvesting. For maximum output power, these base-excited devices are designed to operate at resonance, given that the exciting frequency is constant and sinusoidal. The presence of variable frequencies, multiple frequencies, or non-harmonic excitation has spurred interest in the design of broadband [1], nonlinear [2], and tunable devices that can adapt to these excitation schemes. Useful reviews on techniques to increase the operating bandwidth of energy harvesters are presented by Zhu et al. [3,4] and Twiefel and Westermann [5].

The use of tunable devices, which can adapt to a variable-frequency excitation by changing their natural frequency, has been appealing in situations where the prevailing excitation is harmonic in nature, but exhibits slight changes due to operational conditions or environmental factors. As frequency mismatches lead to a significant reduction in the generated power (off-resonance operation), various tunable devices have been proposed in the literature. For a comprehensive review of frequency tuning methods for piezoelectric energy harvesting systems, the reader is referred to the work of Ibrahim and Ali [6]. The reader is referred to the work of Costanzo and Vitelli [7] for a comprehensive review of recent mechanical tuning techniques for resonant vibration energy harvesters.

In principle, tuning can be achieved by changing the effective mass, stiffness or damping characteristics. Changing the effective mass of a system can be achieved through changing the mass distribution, boundary conditions, or effective dimensions. In this context, reference is made to the work of Gieras et al. [8] in which the effective flexible length of a cantilever arm can be adjusted to change its natural frequency. Efforts to change the system stiffness include the work of Lallart et al. in which a self-tuning scheme for broadband energy harvesting was presented [9], and Lee and Chung [10], in which a rotatable spring with an adjustable spring constant was used to tune the system’s natural frequency between 23–32 Hz.

The use of straining forces to adjust the effective stiffness of a system has also been investigated as a viable approach in tunable devices either by mechanical screws [11] or piezoelectric actuators [12]. One challenge in such devices is to minimize the power needed to provide the straining forces. In practice, this power is taken from the harvested power and must be kept to a minimum if self-sufficient devices are to be designed. This has driven various research efforts utilizing permanent magnets to provide the necessary forces for frequency tuning [13]. Typically, the magnets are moved intermittently by electric actuators or motors in accordance with certain prescribed distances to maintain the system in a state of resonance.

While extensive efforts have been expended on adjusting the stiffness characteristics of structures through the application of straining forces, less emphasis has been placed on adjusting the mass properties. Systems with adjustable mass characteristics are likely to consume less power in tuning, since no mechanical straining forces are to be exerted and maintained, but rather a mass redistribution is needed to cause a change in natural frequency. Devices that require less power for frequency tuning are preferable, as they operate in an environment where energy is already scarce. One of the promising techniques is to use an actuator to reposition masses on the structure, thereby changing the effective moment of inertia through altering the mass distribution. As the only effort needed is to move the position or orientation of a set of discrete masses, energy is kept to a minimum. Efforts oriented along this line include the work of Torbati et al. [14] in which a rotating arm with two sliding masses was used to change the mass moment of inertia of a rotational vibration harvester. Efforts also include previous work by the authors [15] in which the mass moment of inertia of a cantilever beam was adjusted.

In this work, we address this topic by expanding upon the research presented in [15] and proposing to adjust the rotary inertia of a cantilever-type energy harvester in order to enable the device to exhibit a variable resonance frequency, thereby enabling it to operate across a variable frequency range. A mathematical model was developed that couples the cantilever beam with both the variable mass moment of inertia with the harvester. The mathematical model was used to study the effects of the system dimensionless parameters on the tunable response and tunability. Furthermore, an algorithm was developed to self-tune the device to the applied excitation frequency, keeping the system operating at resonance even in the presence of frequency changes. The proposed concept can be applied in real-life applications involving vibration energy harvesting from systems having variable frequencies, such as bridges [16] and sea waves [17], where the natural frequencies are known to depend on the prevailing environment or weather conditions.

## 2. System Description

**Figure 1:**
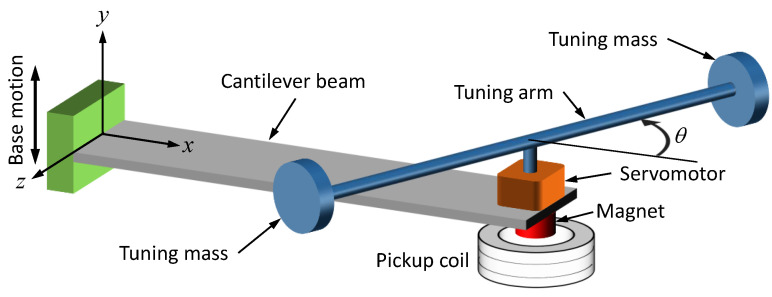
Schematic illustration of variable-inertia device.

The proposed energy harvester consists of a base-excited cantilever beam with a tip mass, as illustrated in Figure 1. The effective mass moment of inertia of the harvester is adjusted through a simple rotatable tuning arm, pinned at the tip of the vibrating cantilever. The tuning arm carries two tuning masses at its tips for better control of the mass moment of inertia. The tuning angle, θ, made between the tuning arm and the *x*-axis varies between 0${}^{\circ }$ and 90${}^{\circ }$ and is controlled by a servomotor that is mounted at the beam’s tip to adjust the rotary inertia of the system about the *z*-axis, thereby effectively varying the natural frequency. A permanent magnet is fixed beneath the motor for electromagnetic harvesting. An electromechanical energy harvesting circuit, composed of a pick-up coil (connected onwards to a load resistance) is placed underneath the magnet.

### 2.1. Dynamic Model

**Figure 2:**
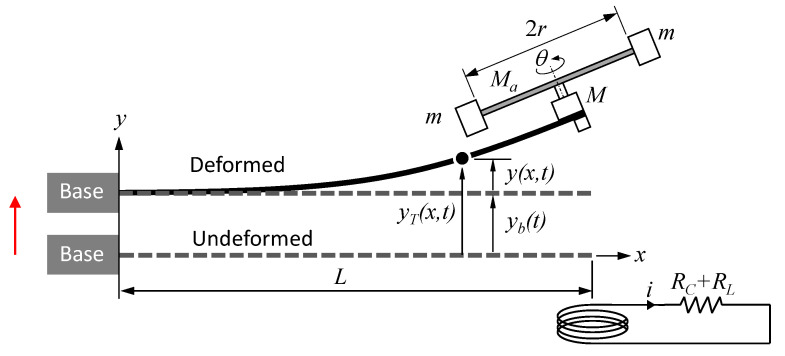
System parameters for analytical modeling.

A mathematical model was developed to predict the effect of the tuning arm angle on the harvester’s fundamental natural frequency, system response, and harvested power. Figure 2 shows the main relevant parameters for the model, where the cantilever beam is represented as a continuous system using the Euler–Bernoulli model of beam bending. The cantilever beam is assumed homogeneous with a length *L*, width *b*, thickness *h*, modulus of elasticity *E*, cross-sectional area *A*, and density ρ. For the sake of simplification, the tuning arm is considered rigid as its second moment of area is much higher than the beam’s. The tuning arm has a mass *M*${}_{a}$, a length 2*r*, and carries two point masses at its ends, each with a mass *m*. The combined mass of the servomotor and magnet is *M*, both considered concentrated masses. Figure 2 illustrates the undeformed and deformed positions of the beam, where a base displacement $y_{b}\left(t\right)$ causes a total displacement of *y*${}_{T}$(*x*,*t*), with *y*(*x*,*t*) being the displacement of the beam relative to the base, i.e.,

(1) $$y_{T}\left(x,t\right)=y_{b}\left(t\right)+y\left(x,t\right).$$

To calculate the system’s fundamental natural frequency as a function of the tuning arm, tuning masses, and their orientation, a free vibration model is first considered.

### 2.2. Free Vibration

For free vibration, we set the base motion to zero ($y_{b}\left(t\right)=$ 0) and express the Euler–Bernoulli equation governing the undamped transverse vibration as:

(2) $$\frac{\partial^{2}y\left(x,t\right)}{\partial t^{2}}+\frac{EI}{\rho A}\frac{\partial^{4}y\left(x,t\right)}{\partial x^{4}}=0.$$

For a system vibrating at its first natural frequency $\omega_{1}$, the general solution of Equation (2) is represented as a harmonic motion at the fundamental frequency $\omega_{1}$:

(3) $$y\left(x,t\right)=Y_{1}\left(x\right)\sin\left(\omega_{1}t\right),$$

where *Y*${}_{1}$(*x*) is the fundamental vibration mode shape, given by:

(4) $$Y_{1}\left(x\right)=\;A_{1}\sinh\left(\beta_{1}x\right)+B_{1}\cosh\left(\beta_{1}x\right)+C_{1}\sin\left(\beta_{1}x\right)+D_{1}\cos\left(\beta_{1}x\right),$$

where β14=ρAω12ρAω12EIEI and *A*${}_{1}$, *B*${}_{1}$, *C*${}_{1}$, and *D*${}_{1}$ are constants that depend on the boundary conditions. At the fixed end of a cantilever beam, we have:

(5) $$\;y\left(0,t\right)=0\;\;,\;\;\frac{\partial y\left(0,t\right)}{\partial x}=0.$$

This produces:

(6) $$Y\left(0\right)=B_{1}+D_{1}=0,$$

(7) $$\frac{dY(0)}{dx}=A_{1}+C_{{}_{1}}=0.$$

The boundary conditions at the free end govern the shearing force and bending moment, and can be written as:

(8) $$\;\;\;EI\frac{\partial^{3}y\left(L,t\right)}{\partial x^{3}}=M_{t}\frac{\partial^{2}y\left(L,t\right)}{\partial t^{2}}+C_{m}\frac{\partial y\left(L,t\right)}{\partial t},$$

(9) $$EI\frac{\partial^{2}y\left(L,t\right)}{\partial x^{2}}=-J\frac{\partial^{3}y\left(L,t\right)}{\partial t^{2}\partial x},$$

where *M*${}_{t}$ is the total mass of the tip components, which includes the mass of the tuning arm (*M*${}_{a}$), tuning masses (2*m*), and the mass of the motor and magnet (*M*), i.e.,

(10) $$M_{t}=2m+M+M_{a}.$$

*C*${}_{m}$ is the damping coefficient of the magnet-coil harvester, and *J* is the effective mass moment of inertia at the cantilever tip, which varies with the rotation angle of the tuning arm θ as:

(11) $$J=2m{\left(r\cos\theta \right)}^{2}+\frac{1}{3}M_{a}{\left(r\cos\theta \right)}^{2},$$

where *r* is the radius of the tuning arm.

The terms on the right-hand side of Equation (8) account for the shearing force due to the inertia of the tip mass, and the electromagnetic force due to the coil-magnet self-inductance. The term on the right-hand side of Equation (9) represents the concentrated bending moment due to the rotary inertia of the tip mass. Substituting Equations (3) and (11) into (8) and (9) yields:

(12) $$\begin{array}{c} A_{1}\left[EI\beta_{1}{}^{3}\cosh\left(\beta_{1}L\right)+\;\left(M_{t}\omega_{1}{}^{2}-C_{m}\omega_{1}\right)\sinh\left(\beta_{1}L\right)\right]\\ +B_{1}\left[EI\beta_{1}{}^{3}\sinh\left(\beta_{1}L\right)+\;\left(M_{t}\omega_{1}{}^{2}-C_{m}\omega_{1}\right)\cosh\left(\beta_{1}L\right)\right]\\ +C_{1}\left[-EI\beta_{1}{}^{3}\cos\left(\beta_{1}L\right)+\;\left(M_{t}\omega_{1}{}^{2}-C_{m}\omega_{1}\right)\sin\left(\beta_{1}L\right)\right]\\ +D_{1}\left[EI\beta_{1}{}^{3}\sin\left(\beta_{1}L\right)+\;\left(M_{t}\omega_{1}{}^{2}-C_{m}\omega_{1}\right)\cos\left(\beta_{1}L\right)\right]=0 \end{array},$$

and

(13) $$\begin{array}{c} A_{1}\left[EI\beta_{1}{}^{2}\sinh\left(\beta_{1}L\right)-\beta_{1}\omega_{1}{}^{2}d^{2}\left(2m+\frac{1}{3}m_{a}\right)\cosh\left(\beta_{1}L\right)\right]\\ +B_{1}\left[EI\beta_{1}{}^{2}\cosh\left(\beta_{1}L\right)-\beta_{1}\omega_{1}{}^{2}d^{2}\left(2m+\frac{1}{3}m_{a}\right)\sinh\left(\beta_{1}L\right)\right]\\ +C_{1}\left[-EI\beta_{1}{}^{2}\sin\left(\beta_{1}L\right)-\beta_{1}\omega_{1}{}^{2}d^{2}\left(2m+\frac{1}{3}m_{a}\right)\cos\left(\beta_{1}L\right)\right]\\ +D_{1}\left[-EI\beta_{1}{}^{2}\cos\left(\beta_{1}L\right)+\beta_{1}\omega_{1}{}^{2}d^{2}\left(2m+\frac{1}{3}m_{a}\right)\sin\left(\beta_{1}L\right)\right]=0 \end{array}.$$

Equations (6), (7), (12) and (13) can be combined to express the system’s characteristic equation in the form:

(14) $$\left[\begin{array}{cccc} 0 & 1 & 0 & 1\\ 1 & 0 & 1 & 0\\ G_{31} & G_{32} & G_{33} & G_{34}\\ G_{41} & G_{42} & G_{43} & G_{44} \end{array}\right]\left[\begin{array}{c} A_{1}\\ B_{1}\\ C_{1}\\ D_{1} \end{array}\right]=\left[\begin{array}{c} 0\\ 0\\ 0\\ 0 \end{array}\right],$$

where $\left[\begin{array}{cccc} G_{31} & G_{32} & G_{33} & G_{34} \end{array}\right]$ and $\left[\begin{array}{cccc} G_{41} & G_{42} & G_{43} & G_{44} \end{array}\right]$ represent the coefficients of *A*_1_, *B*_1_, *C*_1_, and *D*_1_ in Equations (12) and (13), respectively. Solving Equation (14) gives the fundamental natural frequency $\omega_{1}$ as a function of the system parameters, including the tuning angle θ.

### 2.3. Experimental Validation: Free Response

An experimental setup was built consisting of a cantilever beam, the tip mass, and the tuning arm with its tuning masses. Table 1 lists the dimensions and properties of the system. The tuning arm is a solid circular steel rod having a diameter of 3 mm. Compared to the beam, whose thickness is 0.7 mm, the assumption that the tuning arm is rigid (Section 2.1) can be justified. The tuning angle θ was manually changed between 0${}^{\circ }$ and 90${}^{\circ }$ in increments of 10${}^{\circ }$. At each tuning angle, the cantilever was initially disturbed from equilibrium by hand and its free vibration displacement was measured using a laser displacement sensor (Micro-Epsilon OptonCTD 1402, Ortenburg, Germany). The fundamental natural frequency of the beam was investigated for the full tunable range of the tuning arm. A fast Fourier transform (FFT) function was used to analyze the displacement sensor data and determine the system fundamental frequency $\omega_{1}$. The fundamental frequency was also calculated analytically by solving the characteristic Equation (13) using the same set of parameters in Table 1.

**Figure 3:**
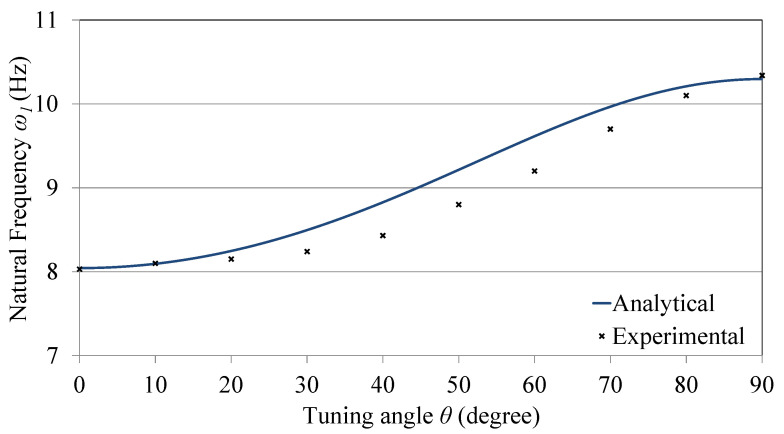
Analytical and experimental natural frequency as a function of the tuning angle.

Figure 3 shows a comparison between the analytical solution of the characteristic equation and the experimentally measured data points. It can be seen that the experimental data match those of the analytical model at both the beginning and the end of the tunable domain; however, there is a small deviation at the mid-range (with a maximum error of 4.7% at $\theta =40^{\circ }$). That is a result of modeling both the tip and tuning masses as point masses, in addition to ignoring the elasticity of the tuning arm.

## 3. Forced Response and Electromechanical Modeling

### 3.1. Forced Vibration

For forced vibration, we impose a steady-state sinusoidal base excitation of the form:

(15) $$y_{b}\left(t\right)=Y_{b}\sin\Omega t,$$

where *Y*${}_{b}$ is the amplitude of base excitation and Ω is the excitation frequency. The governing equation is adjusted to include the effects of base excitation and damping [18,19] and can be written as:

(16) $$EI\frac{\partial^{4}y\left(x,t\right)}{\partial x^{4}}+\rho A\frac{\partial^{2}y\left(x,t\right)}{\partial t^{2}}+C\frac{\partial y\left(x,t\right)}{\partial t}=-\frac{d^{2}y_{b}\left(t\right)}{dt^{2}}\left[\rho A+M_{t}\delta \left(x-L\right)\right].$$

The damping coefficient *C* accounts for both the electromagnetic effect and structural damping, and is given by:

(17) $$C=C_{st}+C_{m},$$

where $C_{st}$ is the structural damping in the system, which was calculated experimentally to be 0.15 N·s/m from measurements of the decay of vibrations, and $C_{m}$ is the electromagnetic damping (see Section 3.3).

According to [20], the presence of tip mass at the cantilever tip creates a jump in the beam distributed mass ρA, which causes the inertial excitation force to be $\rho A+M_{tip}\delta \left(x-L\right)$ to represent the addition of the point tip mass at x=L as proved by the work of Erturk and Inman [18,19].

To obtain the forced response, a single-function approximate solution presented by Leissa [21] can be used. As the excitation force is sinusoidal, the steady-state response can also be assumed to be sinusoidal with same the excitation frequency Ω. The system response can be written as:

(18) $$y\left(x,t\right)=\bar{P}\phi_{1}\left(x\right)\sin\Omega t-\bar{Q}\phi_{2}\left(x\right)\cos\Omega t$$

where $\phi_{1}\left(x\right)$ and $\phi_{2}\left(x\right)$ are trial functions satisfying the boundary conditions of the problem, and $\bar{P}$ and $\bar{Q}$ are their corresponding amplitudes. The function $\phi_{1}\left(x\right)$ is in phase with the forcing function, while $\phi_{2}\left(x\right)$ has a 90${}^{\circ }$ lag from the forcing function. Substituting Equations (17) into (18) produces two separate equations in the form:

(19) $$\begin{array}{cc} \bar{P}EI\frac{d^{4}\phi_{1}\left(x\right)}{dx^{4}}-\bar{P}\rho A\Omega^{2}\phi_{1}\left(x\right)+\bar{Q}C\Omega \phi_{2}\left(x\right) & =F_{0}, \end{array}$$

(20) $$\begin{array}{cc} \bar{Q}EI\frac{d^{4}\phi_{2}\left(x\right)}{dx^{4}}-\bar{Q}\rho A\Omega^{2}\phi_{2}\left(x\right)-\bar{P}C\Omega \phi_{1}\left(x\right) & =0, \end{array}$$

where $F_{0}$ is the excitation force amplitude, represented from Equation (17) as:

(21) $$F_{0}=Y_{b}\Omega^{2}\left[\rho A+M_{t}\delta \left(x-L\right)\right].$$

By assuming that the in-phase deflected shape is the same as the out-of-phase deflected shape, i.e., $\phi_{1}\left(x\right)=\phi_{2}\left(x\right)=Y_{1}\left(x\right)$, the system response represented by Equation (18) can then be written as:

(22) $$y\left(x,t\right)=Y_{1}\left(x\right)\left[\bar{P}\sin\Omega t-\bar{Q}\cos\Omega t\right].$$

For the forcing function $F=F_{0}\sin\Omega t$, Equations (19) and (20) can be approximated using Galerkin integrals [21], so $\bar{P}$ and $\bar{Q}$ can be obtained by solving the equations:

(23) $$\left[\begin{array}{cc} M_{1} & -M_{2}\\ M_{2} & M_{1} \end{array}\right]\left[\begin{array}{c} \bar{P}\\ \bar{Q} \end{array}\right]=\left[\begin{array}{c} F\\ 0 \end{array}\right],$$

where

(24) $$\begin{array}{c} M_{1}=\int_{0}^{L}\left[\left(\frac{EI\partial^{4}y\left(x,t\right)}{\partial x^{4}}-\rho A\Omega^{2}Y\left(x\right)\right)\bar{P}\right]Y\left(x\right)dx,\\ M_{2}=\int_{0}^{L}[-C\Omega \bar{Q}Y\left(x\right)]Y\left(x\right)dx,\;\;Y_{o}\to Y_{b},M_{tip}\to M_{t}\\ F=Y_{b}\Omega^{2}\int_{0}^{L}\left[\rho A+M_{t}\delta \left(x-L\right)\right]Y_{1}\left(x\right)dx, \end{array}$$

where *F* is substituted from Equation (13) as the total force affecting the cantilever.

The total displacement of any point on the beam can then be given by:

(25) $$y_{T}\left(x,t\right)=\left[\bar{P}Y\left(x\right)+Y_{b}\right]\sin\Omega t-\bar{Q}Y_{1}\left(x\right)\cos\Omega t+Y_{b}\sin\Omega t.$$

The vibration amplitude at any point *x* can be calculated by:

(26) $$|Y_{T}\left(x\right)|=\sqrt{{\left(\bar{P}Y\left(x\right)+Y_{b}\right)}^{2}+{\left(\bar{Q}Y\left(x\right)\right)}^{2}}$$

### 3.2. Experimental Validation: Forced Response

The experimental setup described in Section 2.3 was used, where the cantilever was fixed to an Electrodynamic shaker (Brüel & Kjær type 4809, Nærum, Denmark). A neodymium disk magnet (N42) having a diameter of 10 mm and a thickness of 5 mm was attached at its free end (First4Magnets, Tuxford, UK). All system parameters are presented in Table 1. The input signal to the shaker was generated using a function generator (HP 3314A, Hewlett-Packard, Palo Alto, CA, USA) fed to a power amplifier (Brüel & Kjær type 2706, Nærum, Denmark). A pick-up coil of 3.25 mm height and 6.5 mm radius was placed beneath the magnet and the induced voltage through magnet-coil harvester was measured using an oscilloscope (Tektronix TDS 2024C, Beaverton, OR, USA). The beam tip vibration amplitude was measured using a laser displacement sensor.

The energy harvester’s response was investigated both analytically and experimentally at three different tuning angles θ, namely 0${}^{\circ }$, 45${}^{\circ }$, and 90${}^{\circ }$. The angles were chosen to cover the whole tunable span of the device, with angles greater than 90${}^{\circ }$ not considered due to symmetry. The amplitude of base excitation was taken as $Y_{b}=0.2$ mm. Equation (26) was used to calculate the output displacement amplitude *Y*${}_{T}$.

**Figure 4:**
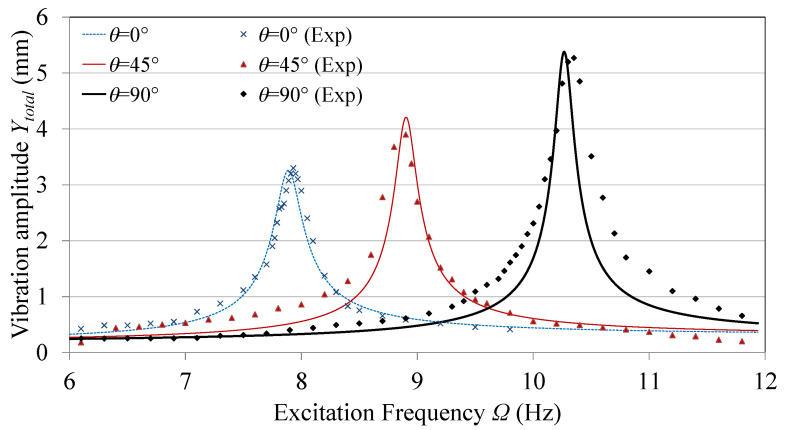
Tip amplitude frequency response.

Figure 4 shows the frequency response of the tip amplitude for different tuning angles, both analytically and experimentally. It can be noted that the system peak frequency increased significantly from 8 to 10.25 Hz with increasing the tuning angle from θ= 0${}^{\circ }$ to 90${}^{\circ }$. This observation is in accordance with that in Figure 3. The vibration amplitude also increases with the increase in the excitation frequency as the effective excitation force increases along with the excitation frequency, as proved by Equation (21). That is also in addition to the drop of the effective moment of inertia of the beam tip when the tuning arm gets closer to 90${}^{\circ }$. It can be noted that the analytical predictions and the experimental results agree with minimum deviations. Deviations between the analytical and experimental results can be attributed to the point-mass assumptions of the tip mass and tuning masses, in addition to neglecting the flexibility of the tuning arm, which affect the value of natural frequency and damping. While no torsional vibrations were considered in the model, slight torsional vibrations in the experiments are inevitable, despite efforts to keep the tuning arm symmetric. Such vibration modes are likely to change the system dynamics.

### 3.3. Electrical Output

As the operational excitation frequencies are low, the coil inductance can be neglected [19,20]. The induced voltage can be obtained from Faraday’s law as:

(27) $$V\left(t\right)=-Bl_{c}v_{t}\left(t\right),$$

where *V*(*t*) is the induced voltage across, *B* is the magnetic flux density, *l*${}_{c}$ is the total coil wire length, and *v*${}_{t}$(*t*) is the velocity of the magnet in the transverse direction, calculated from Equation (24) at the cantilever tip (x=L) as:

(28) $$v_{t}\left(t\right)=\frac{\partial y_{T}\left(L,t\right)}{\partial t}=\Omega \left[\left(\bar{P}Y\left(L\right)+Y_{b}\right)\cos\Omega t+\bar{Q}Y\left(L\right)\sin\Omega t\right].$$

From Equations (26)–(28), the amplitude of the induced voltage can be expressed as:

(29) $$\left|V\right|=-Bl_{c}\left|v_{t}\right|=-Bl_{c}\Omega \left|Y_{T}\left(L\right)\right|.$$

The coil resistance *R*${}_{c}$ is the main effective component in the coil impedance especially for low excitation frequencies [22]. A resistive load *R*${}_{L}$ is connected across the coil ends to allow a current *i*(*t*) to flow in the circuit such that:

(30) $$i\left(t\right)=\frac{V}{R_{L}+R_{C}}.$$

The harvested power *P* can be then expressed as:

(31) $$P=i^{2}\left(t\right)R_{L}=\frac{V^{2}}{{\left(R_{C}+R_{L}\right)}^{2}}R_{L}.$$

Substituting Equation (29) into (31) yields the power as:

(32) $$\left|P\right|=\frac{{\left(Bl_{c}\right)}^{2}R_{L}}{{\left(R_{L}+R_{C}\right)}^{2}}{\left[\Omega |Y_{T}\left(L\right)|\right]}^{2}.$$

The current *i*(*t*), induced in the coil, creates a reverse magnetic field that creates an opposing electromagnetic force $F_{m}$ on the magnet as:

(33) $$F_{m}=l_{c}Bi\left(t\right)=\frac{{\left(l_{c}B\right)}^{2}}{R_{L}+R_{C}}v_{t}\left(t\right)=C_{m}v_{t}\left(t\right),$$

where *C*${}_{m}$ is the electromagnetic damping coefficient given by:

(34) $$C_{m}=\frac{{\left(l_{c}B\right)}^{2}}{R_{L}+R_{C}}$$

### 3.4. Harvested Power

**Figure 5:**
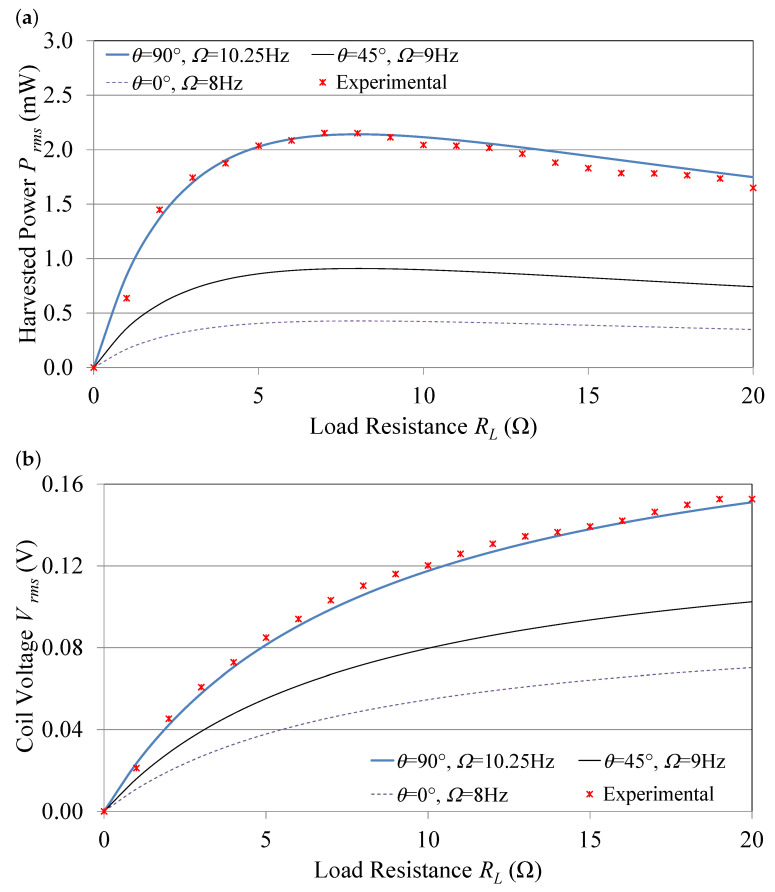
(**a**) Output voltage and (**b**) harvested power.

The effect of the load resistance *R*${}_{L}$ on the power *P* and the induced voltage *V* was investigated. *V* and *P* were calculated using Equations (29) and (31), respectively, at three different tuning angles θ, namely 0${}^{\circ }$, 45${}^{\circ }$, and 90${}^{\circ }$. Figure 5a,b show the analytical results, which were validated experimentally at the 90${}^{\circ }$ tuning angle on the experimental setup described in Section 3.2 at an excitation frequency of 10.25 Hz, which corresponds to the resonance frequency at θ= 90${}^{\circ }$. A resistance box was used to change *R*${}_{L}$ from 1 Ω to 10 MΩ. The voltage across load was measured using an oscilloscope.

The figures show a good matching between experimental results and analytical curves, which validates the analytical model. Figure 5a shows that the harvested power value starts to increase with *R*${}_{L}$ until it reaches its peak and then starts decreasing. Here it shows that the maximum RMS power harvested corresponded to a load resistance of 8 Ω. Thus, an optimum load resistance *R*${}_{L}=$ 8 Ω needs to be selected to maximize the harvested power from this device. Figure 5b shows that induced voltage across the coil ends (*V*) starts from zero with no load resistance and increases with the increase in load resistance. The induced voltage increases with the excitation frequency. This is due to the increase in the magnet’s speed, which leads to a higher rate of magnetic flux change inside the pick-up coil.

### 3.5. Parametric Analysis

**Figure 6:**
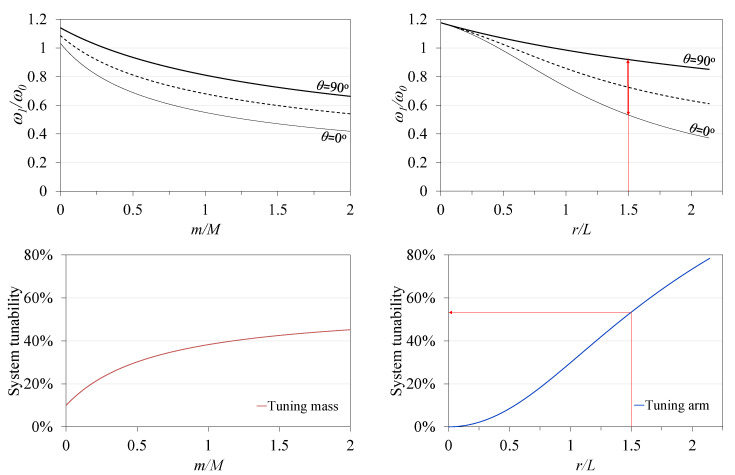
Generic dimensionless parameter effects on system tunable response and tunability.

To gain insight into the effect of the tuning arm length (*r*) and tuning mass value (*m*) on the tunable frequency domain, a dimensionless analysis was performed and normalized to the resonance frequency $\omega_{0}$, which is defined as the resonance frequency of the current system after treating the tuning arm and tuning masses as concentrated (point) masses placed at the beam tip. The tuning arm length (*r*) is referred to the main cantilever length (*L*), and the tuning mass (*m*) is referred to the tip mass (*M*). The effect of *r/L* and *m/M* on the system tunability can be seen in Figure 6. The system tunability can be defined as the ratio between the width of the tunable span and its average value. Figure 6 shows an example where the ratio *r*/L yielded a tunable span of $\Delta \omega_{1}=0.39\omega_{0}$, resulting in system tunability of 53.4%. The tuning arm length has a significant effect on the system tunability. Despite the greater tunability offered by longer arms, issues such as the arm’s flexibility and bending moments reflected on the servomotor shaft impose practical limitations on the use of excessively long arms.

## 4. Self-Tuning

**Figure 7:**
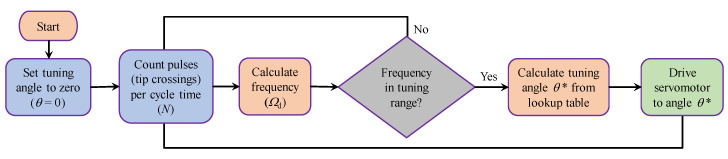
Flowchart illustrating the tuning algorithm.

An algorithm was developed herein to self-tune the device to the applied excitation frequency, thereby keeping the system operating at resonance. The structure of the tuning algorithm relies on (a) measuring the frequency at which the beam is vibrating (excitation frequency, Ω), (b) comparing this frequency with the natural frequency ($\omega_{1}$) corresponding to the current angular position of the tuning arm (θ), and (c) driving the servomotor to the desired tuning angle to achieve resonance. This process is schematically illustrated in Figure 7 and is repeated cyclically using a microcontroller device (Arduino Uno, Boston, MA, USA) to allow for continuous frequency adjustments. The prevailing frequency is measured experimentally using a photo-interrupter sensor (type OS25B10), which is an infrared sensor/receiver module positioned beside the beam tip. As the beam vibrates, its tip interrupts the sensor light producing a voltage pulse. A microcontroller is programmed to count the number of pulses in a certain given time, thus calculating the frequency of vibration.

The tuning procedure starts by setting the tuning arm to its zero-degree position, aligned with the cantilever axis, when the system is switched on. First, the control algorithm senses the cantilever oscillations using the photo-interrupter sensor, then the input excitation frequency is detected and calculated. The frequency is checked to ensure it is in the range domain defined for the device; if not, the frequency detection is repeated. If the frequency is in the tunable range, the microcontroller calculates the corresponding tuning angle needed for this specific frequency via a lookup table, based on the data presented in Figure 3, to keep the system in a resonance state. The servomotor then rotates the tuning arm to the calculated position for resonance, and the process is repeated as long as the controller is on.

The employed frequency detection technique relies on counting the number of times (*N*) the oscillating cantilever tip crosses its equilibrium position during a specific detection time (*D*${}_{int}$). The detected frequency ($\Omega_{d}$) can thus be calculated as $\Omega_{d}=N/\left(2D_{int}\right)$. Changing the detection interval *D*${}_{int}$ changes the sensor’s resolution ($f=1/(2D_{int})$), defined as the smallest frequency that can be recognized. For higher resolution measurements, a longer *D*${}_{int}$ is required, which might not be effective for applications with excitation frequencies changing at a fast rate. Short detection intervals enable the control system to adapt to fast-changing excitation frequencies, however, resulting in less control resolution and reduced accuracy in the frequency measurements.

Experiments were conducted using the self-tuning system to display the effect of the detection interval *D*${}_{int}$ on the accuracy of the excitation frequency measurements, thus, the tuning of the system. The experiment starts with the system tuned to the input excitation frequency ($\omega_{1}=\Omega_{1}$). The excitation frequency is then changed from its initial value Ω${}_{1}$ to Ω${}_{2}$, causing the system to be out of resonance ($\omega_{1}\neq \Omega_{1}$). The number of times (*N*) the oscillating cantilever tip crosses its equilibrium position during a specific detection time (*D*${}_{int}$) is counted by the microcontroller and $\Omega_{d}$ is calculated. The system is tuned to $\omega_{1}=\Omega_{d}\approx \Omega_{2}$, attempting to bring the system back to the resonance state. How close $\Omega_{d}$ is to $\Omega_{2}$ depends on the sensor’s resolution *f*, which improves as $D_{int}$ increases. As a proof of concept, the input excitation frequencies $\Omega_{2}$ were arbitrarily selected as 8.4 Hz, 8.875 Hz, and 9.185 Hz.

**Figure 8:**
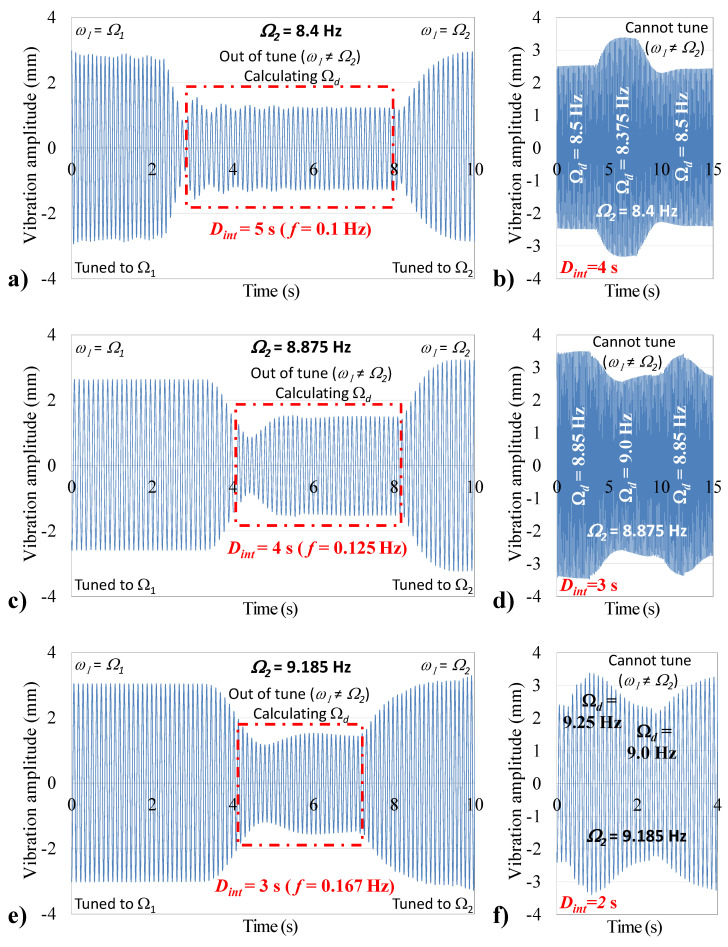
Effect of detection interval *D*${}_{int}$ on system response at *x = L*, presented at (**a**) $\Omega_{2}=$ 8.4 Hz and $D_{int}=$ 5 s, (**b**) $\Omega_{2}=$ 8.4 Hz and $D_{int}=$ 4 s, (**c**) $\Omega_{2}=$ 8.875 Hz and $D_{int}=$ 4 s, (**d**) $\Omega_{2}=$ 8.875 Hz and $D_{int}=$ 3 s, (**e**) $\Omega_{2}=$ 9.185 Hz and $D_{int}=$ 3 s, and (**f**) $\Omega_{2}=$ 9.185 Hz and $D_{int}=$ 2 s.

Figure 8a shows a tuned system, where the input excitation frequency was changed to $\Omega_{2}=8.4\;\mathrm{Hz}$. Selecting a detection interval *D*${}_{int}=$ 5 s (f= 0.1 Hz) enabled the controller to correctly measure $\Omega_{d}=\Omega_{2}$, enabling the tuning system to achieve resonance ($\omega_{1}=\Omega_{2}$). Selecting the alternative detection interval *D*${}_{int}=$ 4 s reduced the resolution to f= 0.125 Hz, thus, only enabling the controller to calculate the frequencies nearest to $\Omega_{2}$ that are multiples of *f*, i.e., 8.5 Hz and 8.375 Hz, as shown in Figure 8b. Similarly, Figure 8c,d show the system successfully tuning to input excitation frequencies 8.875 and 9.185 Hz when using detection intervals of 4 s and 3 s, respectively. Figure 8d,e again show failures to correctly calculate $\Omega_{2}$ when using *D*${}_{int}$ of 3 s (f= 0.167 Hz), and 2 s (f= 0.25 Hz), respectively, which again resulted in the system tuning to one of its frequencies nearest to $\Omega_{2}$ that are multiples of the resolution *f*.

## 5. Conclusions

The proposed tunable energy harvester introduces a new tuning approach of having a variable mass moment of inertia. The harvester can function in a wide domain to follow the changing excitation frequency by changing the tuning angle from zero to ninety degrees. For selected system scale and dimensions, the tunable domain is [8:10.25] Hz with a resolution of 0.1 Hz, which makes the harvester capable of smooth tuning. On this scale, the device tunability reaches 25% and can be increased, as shown in parametric analysis, by a proper selection of the tuning arm length and tuning mass value. The tuning algorithm used makes a closed repeating loop that has a detection interval of 5 s to respond to the changing excitations. The algorithm calculates the excitation frequency before tuning and compares it with the domain limits. This step overcomes the pulsations in frequency due to readings errors and keeps tuning going smoothly.

The servomotor used to rotate the tuning arm is self-locked, which allows the system to be intermittently controlled as power is only used to rotate the tuning arm with no power needed to maintain it in place. The proposed system consumes power only to overcome the internal motor friction, as no other mechanical work is required by the motor. With intermittent tuning, no mechanical forces are required once the motor is set in a certain position, unlike systems that require a continuous application of straining forces. The harvester used is a coil-magnet harvester that generates power by induction and it was selected in this research for its low working frequencies. Power can be also harvested by attaching a piezoelectric strip to the main beam, but this idea will be more effective for high-frequency applications.

## Figures and Tables

**Table 1:** System parameters.

Parameter	Value	Parameter	Value
*L*	140 mm	*r*	125 mm
*b*	28 mm	*m*	5 g
*h*	0.7 mm	$r_{arm}$	3 mm
$C_{st}$	0.15 N·s/m	$m_{arm}$	12 g
*E*	213 GPa	*M*	16.5 g
ρ	7850 kg/m^3^	*B*	1.3 T
*R* ${}_{C}$	8 Ω	*R* ${}_{L}$	8 Ω

## Data Availability

Not applicable.
